# Tibial torsion analysis in computed tomography: development and validation of a real 3D measurement technique

**DOI:** 10.1186/s13244-020-00960-w

**Published:** 2021-02-15

**Authors:** Armando Hoch, Tabitha Roth, Magda Marcon, Philipp Fürnstahl, Sandro F. Fucentese, Reto Sutter

**Affiliations:** 1 Department of Orthopaedics, Balgrist University Hospital, University of Zurich, Forchstrasse 340, 8008 Zurich, Switzerland; 2 Research in Orthopaedic Computer Science, Balgrist University Hospital, University of Zurich, Zurich, Switzerland; 3 Balgrist University Hospital, University of Zurich, RadiologyZurich, Switzerland

**Keywords:** Tibial torsion, 3D measurement, Lower-limb deformity, Computed tomography, Torsional malalignment

## Abstract

**Purpose:**

Pathological tibial torsion is known to negatively influence the functionality of the lower extremity, and therefore, its assessment might play an important role. While 3D imaging is used for many examinations of the musculoskeletal system, for the determination of tibial torsion no 3D measurement technique has been available so far. We developed a 3D measurement method and assess its interobserver reliability as well as its correlation with standard 2D measurement methods.

**Methods:**

CT scans of 82 tibiae in 79 patients with a mean age of 41 years were included. A novel 3D measurement technique was developed and applied. Measurements were compared with two frequently used 2D measurement methods. ICC (intraclass correlation coefficient) for the new technique was determined and compared to the 2D measurement method. Furthermore, differences between left and right legs as well as between males and females were assessed.

**Results:**

The ICC for the 2D methods was 0.917 and 0.938, respectively. For the 3D measurements, ICCs were calculated to be 0.954 and 0.950. Agreement between 2 and 3D methods was moderate to good with ICCs between 0.715 and 0.795. Torsion values for left and right legs did not differ significantly in 2D and in 3D (26.2 vs 28.5° and 27.2 vs. 25.9°). The same is true for the differences between male and female in 2D and 3D (26.2 vs. 29.6° and 25.0 vs. 31.2°).

**Conclusion:**

The newly developed 3D measurement technique shows a high intraclass agreement and offers an applicable opportunity to assess the tibial torsion three-dimensionally.

## Key messages

- The tibial torsions relevance in maltorsion of the lower extremity and respective surgical planning is underestimated.
- So far, no real 3D measurement technique in sectional imaging was elaborated.
- Our novel 3D measurement technique showed an excellent intraclass correlation.

## Introduction

Rotation of the tibia along its longitudinal axis was first described in an anatomical study in 1903 by Pierre Germain Marie Le Damany, who was a professor for anatomy and physiology, famous for his early work on the developmental dysplasia of the hip (DDH) [1]. According to him, excessive tibial torsion can be understood—like DDH—as element of the 'packing disorders' that can arise during the development and growth of the fetus in utero. Elements of his pioneering work remain true to this day [1–3]. The term 'tibial torsion' was then consolidated in a radiographic study in 1949 [4]. Since the tibial torsion is subject to major changes during childhood, most studies have focused on its normal and pathological development in this early phase of life [5–10]. Decreased or increased tibial torsion was found to be associated with the clinical picture of in- or out-toeing, which in turn is associated with a higher risk for stumbling, gait abnormalities and aesthetic complaints [7, 8, 10]. In adults, the tibial torsion can be pathologically altered after trauma and consecutive surgery (e.g., intramedullary nails), sometimes leading to an inferior clinical outcome [11–15], such as a reduced capacity of muscles to extend the hip and knee joints in patients with excessive tibial torsion [16]. Furthermore, rotational deformities of the lower extremity in general are associated with a higher risk for osteoarthritis of the involved joints [17–21]. Surgical correction due to these issues is sometimes necessary, and therefore, a validated measurement method for the tibial torsion is required. Radiographic measurement methods using standard radiographs [4, 9] or fluoroscopy [22] have proven to be inadequate, since their reproducibility is low. With the emerging possibilities in cross-sectional imaging, different computed tomography (CT)-based 2D measurement methods were proposed [23–28]. In contrast to the femur however, a clear definition of the joint axes is difficult for the tibia. On the one hand, the determination of the proximal axis for 2D measurements, the highly variable posterior shape of the proximal epiphysis and metaphysis is a challenge for routine measurements. On the other hand, the distal axis, as defined by most standard methods, is directly dependent on the highly variable position of the fibula [24]. Various attempts have been undertaken to assess 2D measurements of tibial torsion, but the variabilities between some of these methods remain substantial [23–28]. Thus, the role of the tibial torsion in lower extremity deformities remains unclear, and with the increasing demand of a holistic 3D deformity analysis, a reliable measurement technique for the tibial torsion is mandatory. To our knowledge, there is no measurement technique which considers the three-dimensional (3D) constitution of the tibia using sectional imaging, and also the relevance of the position of the fibula for calculation of the tibial torsion has never been evaluated. Therefore, the first purpose of this study was to develop and validate a 3D measurement technique to assess tibial torsion. The second purpose was to investigate the correlation between by the most frequently used 2D measurement methods with the newly developed 3D technique.

## Material and methods

### Patient selection

We retrospectively identified 79 consecutive patients who received a CT scan of one or both legs. The data acquisition was performed using the MyOsteotomy CT protocol (Medacta, Castel San Pietro, Switzerland) in which only the hip, knee and ankle joints were scanned, while skipping midshaft regions. All CT scans were performed at our institution between 03/2016 and 12/2018. These patients obtained the CT scan in the scope of the preoperative planning for lower-limb surgical procedures (high tibial osteotomy in unicompartmental osteoarthritis of the knee) using patient-specific instruments. Three patients with prior osseous surgery on the tibia were excluded subsequently in order to obtain a collective without iatrogenic alteration of bony anatomy. Finally, we included CT scans of 82 knees from 79 patients (57 males and 22 females, 49 left and 33 right legs) with a mean age of 41 years (range 15–64 years). The local ethical committee approved this study (Zurich Cantonal Ethics Commission, 2017-01,616), and all patients gave their informed consent for the use of their data for research purposes.

### CT examinations and segmentation

All CT scans were performed at our institution, using a 64-detector row CT scanner (Somatom Definition AS Siemens Healthcare, Erlangen, Germany). Slice thickness was 1.0 mm with an in-plane resolution of 0.4 × 0.4 mm. The CT scans were segmented using the global thresholding and region growing functionality of a standard segmentation software (Mimics Medical, Materialise NV, Leuven, Belgium) in order to generate 3D bone models [29–31].

### 3D measurement methods

**Fig. 1:**
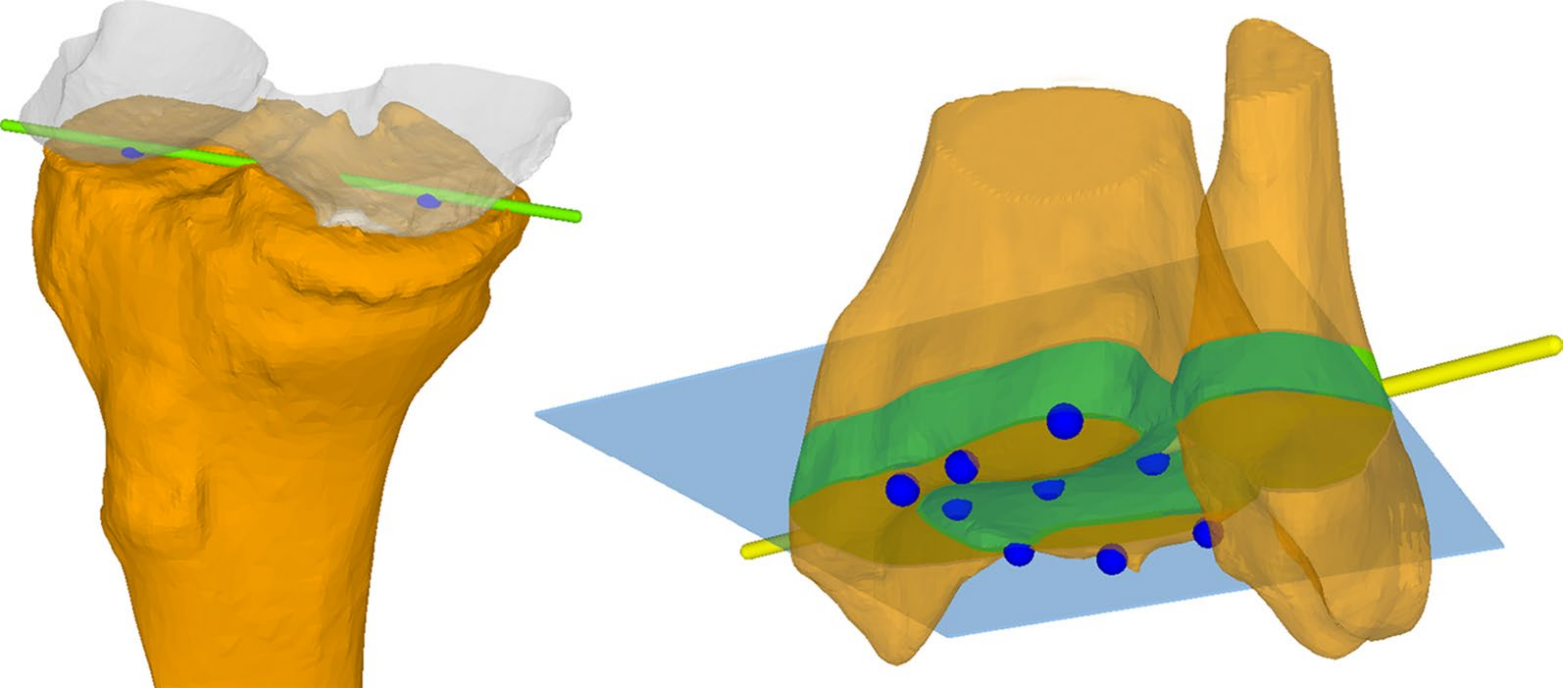
Definition of the proximal and distal tibia axes in 3D shown on a model of the proximal tibia (orange) and distal femur (transparent grey) on the left and the distal tibia and fibula (both orange) on the right side. Left: the most distal points were identified for each femoral condyle, and PCA was applied to the corresponding points on the tibial plateau (blue dots). The PTA was defined as the first principle component. Right: A plane (DTJP, blue) was fitted to nine manually selected points on the articular surface (blue dots). A bone slice of 10 mm thickness was extracted distal from the DTJP (green), and PCA was applied to find the DTA (yellow line). DTA was defined as the second principle component. The DTA was calculated in two different adaptations (with and without consideration of the fibula). *PCA* principle component analysis, *PTA* proximal tibia axis, *DTJP* distal tibia joint plane, *DTA* distal tibia axis

Tibial torsion in 3D was defined as the angle between the proximal (PTA) and the distal tibia axis (DTA), projected to the plane perpendicular to the anatomical axis of the tibia (Fig. 1). While the PTA was the same for all measurements in one case, the DTA was calculated twice for each bone: once by considering only the distal tibia itself (3D-T) and once by additionally including the distal fibula (3D-TF).

Prior to the definition of the PTA and DTA, the bone models have to be aligned in a reference coordinate system. The mechanical axis, determined as described by Fürnstahl et al. [32], was aligned with the y-axis, and the leg was rotated until the patella was facing anteriorly.

The PTA was defined by a line connecting the points on the tibial plateau that are closest to the most distal tips of the femoral condyles. In a first step, the two condyles were automatically detected by applying a k-means algorithm [33] to the most distal 25 mm of the femoral bone, initiated as described by Arthur et al. [34]. Two condylar tip clusters (CTC) were defined as all points within the most distal 1 mm of each condyle. Subsequently, the closest point on the tibial plateau was found for each point within the CTCs, yielding the tibiofemoral contact areas (TFCA) on the tibial plateau. Finally, principal component analysis (PCA) was applied to the TFCA, defining the PTA [35].

In order to determine the DTA, the distal tibia joint plane (DTJP) was fitted to the articular surface of the distal tibia by applying a least square plane fit algorithm to nine manually selected points (Fig. 1) [35]. We then isolated the 10 mm of tibial metaphysis located directly proximal of the DTJP and used it to define the DTA by applying PCA [35]. For the determination of the 3D-TF tibial torsion, the corresponding 10 mm of fibular metaphysis was additionally included.

Both PTA and DTA were finally projected onto a plane perpendicular to the anatomical axis of the tibia. The anatomical axis was defined by fitting a line through the center points of six intersections along the tibia.

These points for the anatomical axis were found by first fitting a plane to the tibial plateau by applying a least square fit algorithm [35] to eight manually selected points on the plateau, resulting in the tibial plateau plane (TPP). Thereafter, PCA was applied to the whole tibial bone. The first principal component was used as the plane normal for six parallel planes placed along the tibial metaphysis and proximal shaft in 20-mm intervals, starting 40 mm distal from the center of the TPP. For each of these planes, the intersection with the tibial bone was calculated, and the center point of each intersection was determined. Finally, the anatomical axis was fit through these six center points.

### 2D measurement methods

**Fig. 2:**
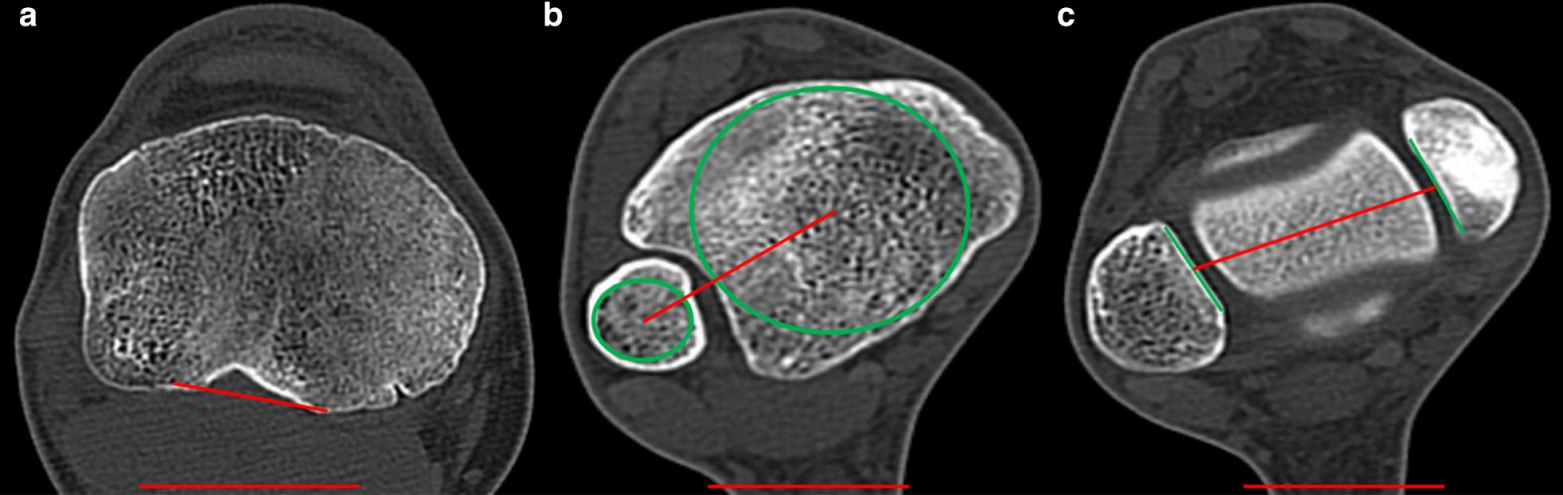
2D measurement methods (**a**) proximal determination for both techniques (angle between red lines), (**b**) distal determination for technique 1, (**c**) distal determination for technique 2.—2D tibial torsion measures are calculated by the sum of angle between red lines in **a** plus angle between red lines in **b** for technique 1 and in **c** for technique 2

Two frequently used 2D measurement techniques were chosen for comparison to the proposed 3D technique (Fig. 2). Both techniques used the same approach to determine the orientation of the PTA. A tangent was placed along the posterior cortex of the tibial head one slice (1.0 mm) proximal to the fibular head. The approach to determine the DTA differed between the two techniques. For the technique according to Jakob et al. [23, 26], two circles were drawn in the slice (1.0 mm) proximal to the articular surface of tibia in the ankle joint, one circle was fitted into the tibia and one into the fibula in order to determine the respective centers. The DTA was drawn as a line connecting the two centers. For the technique according to Goutallier et al. [25], two tangents were placed to the articular aspects of the medial and the lateral malleolus one slice (1.0 mm) below the talar surface. The DTA was drawn as the line connecting the centers of the two tangents. For both techniques, the tibial torsion was calculated as the angle between the PTA and the DTA. All measurements were performed independently by one orthopedic surgeon and one radiologist (A.H. and M.M.).

### Statistical analysis

All data were documented with the REDCap software (Research Electronic Data Capture, Vanderbilt University, Nashville, Tennessee, USA). Statistical analyses were performed with SPSS (IBM SPSS Statistics 25, IBM, Armonk, New York, USA). Interobserver agreement was determined by calculating the intraclass correlation coefficients (ICC) based on a two-way random effects model assessing absolute agreement of single measures between readers. Agreement between different measurement methods was assessed using the ICC based on a two-way mixed model for absolute agreement of average measures generated. Differences of means between different methods were compared using paired samples t test. Measurements for left and right legs as well as male and female patients were compared by applying independent samples t tests to the means of the 2D and 3D methods, respectively. All data used for t tests were tested for normality with a Shapiro–Wilk test. Mean absolute differences (MAD) were calculated. Tests were evaluated using a significance level of *p* ≤ 0.05.

## Results

### Interobserver Agreement

Results for interobserver agreement are summarized in Table 1. The ICC for the Jakob and Goutallier methods were 0.917 (95% CI 0.755–0.962) and 0.938 (95% CI 0.905–0.959), respectively. For the 3D measurements, ICCs were 0.954 (95% CI 0.930–0.970) and 0.95 (95% CI 0.924–0.968) for the 3D-TF and 3D-T methods, respectively.

**Table 1:** Correlation coefficients of interobserver agreement for all four methods

Interobserver agreement 2D	Jakob method	Goutallier method
ICC (95% CI)	0.917 (0.755–0.962)	0.938 (0.905–0.959)

Interobserver agreement 3D	3D-TF	3D-T
ICC (95% CI)	0.954 (0.963–0.985)	0.950 (0.924–0.968)

*ICC* intraclass correlation coefficient, *CI* confidence interval, *3D-TF* 3D method considering tibia and fibula, *3D-T* 3D method only considering tibia

### Intermethod Agreement

All data were normally distributed. ICCs for intermethod agreement are summarized in Table 2. Agreement between the 2D methods was good (0.873 (95% CI − 0.115–0.968)), while 3D methods had excellent agreement (0.950 (95% CI 0.860–0.977). Means were significantly different for both, 2D (30.1 vs. 24.1° for Jakob and Goutallier, respectively, *p* < 0.001, mean difference 6 ± 2.6°, range 0.3–12.7°) and 3D methods (28.1 vs. 25.2° for 3D-TF and 3D-T, respectively, *p* < 0.001, mean difference 2.9 ± 4.5°, range − 12.0–10.2).

**Table 2:** Intermethod agreement for all combinations between the four tested methods

Intermethod agreement	ICC (95% CI)	Mean value, *t* test
Jakob vs. Goutallier	0.996 (0.994–0.998)	30.1 vs. 24.1° (*p* < 0.001) MD: 5.9 ± 2.6° (range 0.3–12.7°)
Jakob vs. 3D-TF	0.783 (0.684–0.853)	30.1 vs. 28.1° (*p* = 0.033) MD: 2.0 ± 8.5° (range − 23.5–24.6°)
Jakob vs. 3D-T	0.715 (0.481–0.834)	30.1 vs. 25.2° (*p* < 0.001) MD: 4.9 ± 9.6° (range − 16.4–26.3°)
Goutallier vs. 3D-TF	0.795 (0.681–0.868)	24.1 vs. 28.1 (*p* < 0.001) MD: − 3.9 ± 8.7° (range − 31.6–16.6°)
Goutallier vs. 3D-T	0.729 (0.581–0.825)	24.1 vs. 25.2 (*p* = 0.348) − 1.0 ± 9.9° (range − 24.5–19.9°)
3D-TF vs. 3D-T	0.950 (0.860–0.977)	28.1 vs. 25.2° (*p* < 0.001) MD: 2.9 ± 4.5° (range − 10.2–12.0°)

*ICC* intraclass correlation coefficients, *CI* confidence interval, *MD* mean difference

Agreement between 2 and 3D methods was moderate to good for all combinations. The Jakob method had ICCs of 0.795 (95% CI 0.681–0.868) and 0.715 (95% CI 0.481–0.834) when compared with 3D-TF and 3D-T, respectively. The Jakob method mean was also significantly different to both 3D-TF and 3D-T (30.1 vs. 28.1° (*p* = 0.033), mean difference 2.0 ± 8.5° (range − 23.5–24.6°) and 30.1 vs. 25.2° (*p* < 0.001), mean difference 4.9 ± 9.6° (range − 16.4–26.3°)).

The ICCs for the Goutallier method were calculated to be 0.746 (95% CI 0.558–0.847) and 0.729 (95% CI 0.581–0.825). Its mean was significantly different to the 3D-TF method (24.1 vs. 28.1 (*p* < 0.001), mean difference − 3.9 ± 8.7° (range − 31.6–16.6°)), but not to the 3D-T method (24.1 vs. 25.2 (*p* = 0.348), mean difference − 1.0 ± 9.9° (range − 24.5–19.9°)).

### Left vs. Right and Female vs. Male

Results regarding differences between side and genders are summarized in Table 3. All data were normally distributed. Torsion values for left and right legs did not differ significantly in 2D as well as in 3D (26.2 ± 8.2 vs 28.5 ± 9.1° (*p* = 0.232) and 27.2 ± 12.8 vs. 25.9 ± 10.6° (*p* = 0.632)). The same is true for the differences between male and female in 2D (26.2 ± 8.7 vs. 29.6 ± 8.0° (*p* = 0.115)); it was however significantly different when assessed in 3D (25.0 ± 10.8 vs. 31.2 ± 13.7° (*p* = 0.036)).

**Table 3:** *t* tests for side and gender differences of tibial torsion values

Left vs. right	2D	3D
*t* test (*p* value)	26.2 ± 8.2 vs. 28.5 ± 9.1° (*p* = 0.232)	27.1 ± 12.8 vs. 25.8 ± 10.5° (*p* = 0.632)

Male vs. female	2D	3D
*t* test	26.2 ± 8.7 vs. 29.6 ± 8.0° (*p* = 0.115)	25.0 ± 10.8 vs. 31.2 ± 13.7° (*p* = 0.036)

## Discussion

Rotational malalignment of the lower extremities has received considerable attention in the last two decades, not only in children, but also in adults. However, the vast majority of studies have focused on abnormal femoral torsion and its role in femoroacetabular impingement, developmental dysplasia of the hip, patellofemoral disorders and other diseases [36–42]. Maltorsion of the tibia in adults remains poorly investigated. Nevertheless, there is certain knowledge about the negative clinical outcome in patients with a decreased or increased tibial torsion [11–16]. In addition, the lack of standardized measurement methods may have resulted in too little attention being paid to the relevance of tibial torsion.

The most important finding of this study is that the newly developed three-dimensional measurement technique showed a high interobserver reliability. The technique is capable of delivering values for the tibial torsion both with or without consideration of the fibula position.

The interobserver reliability of the 2D measurement techniques was slightly lower than for the 3D methods, achieving similar values as in the current literature [27]. The interclass correlation coefficients comparing the 3D technique with the two 2D measurement techniques for the tibial torsion were only moderate to good. However, a lower ICC does not necessarily implicate limited suitability of the methods. In this case, the differences might be explained by the diverging definition of the proximal tibial axis in the proposed 3D techniques. The 2D measurement methods define the proximal axis as the tangent to the dorsal cortex of the tibial head, but the kinematic relevance of the dorsal rim of the tibial bone is questionable. The 3D technique proposed in our paper, on the other hand, uses the part of the tibial plateau which is in contact with the femoral condyles to define the PTA. Since a holistic 3D analysis with consideration of the kinematically relevant structures is desirable, we believe that this is a more suitable representation of the proximal tibial axis.

Since cross-sectional imaging has become standard in preoperative assessment of many orthopedic procedures, several studies were conducted to describe techniques for torsion measurements of the tibia based on 2D CT or MR images [23–28]. All proposed methods still used a two-dimensional technique applied on selected slices at predefined anatomical positions of the tibia. None of them considers the three-dimensional anatomical constitution of the tibia. Fürmetz et al. proposed a three-dimensional assessment of the lower extremity as a whole [43]. In their work, tibial torsion was determined by the two most dorsal proximal points of the tibia and the two outermost points at the height of the ankle joint, a technique adapted from Liodakis et al. [27]. Even if calculated on the basis of three-dimensional landmark coordinates, their measurement technique only uses four points to define the torsion of a volumetric body. An interesting approach was proposed by Borish et al. where tibial torsion was measured using a capture motion system [44]. This is valuable with the increasing possibilities for dynamic analyses. However, some inaccuracy remains as the system uses surface markers that do not accurately reflect the anatomical landmarks of the tibia. The system is also not available in many facilities. However, we think that such a complex problem requires a technique which considers the whole three-dimensional surface of the tibial bone. Our method meets this requirement since a volumetric model is used for the calculation and it is therefore closer to the anatomic reality than points selected by the examiner. The fact that the method works with or without consideration of the fibula is convenient, since the position of the fibula is variable [24]. However, since the fibula is a relevant stabilizer for the ankle joint and makes a relevant contribution to the ankles kinematic function, we think it should be included in the analysis. However, the fibula can be excluded if it shows posttraumatic or postoperative alterations that make it unreliable.

A limitation of our study is that CT scans are not a first line diagnostic tool in every patient and segmentation is time-consuming and requires specific knowledge, which is not always accessible. However, modern CT technology has resulted in a substantially lower radiation dose, and future algorithms will be able to automatically apply 3D measurement methods to segment 3D models. 3D measurement methods, like the one presented in this paper, will be increasingly implemented in the surgery planning workflow in the coming years. Moreover, a robust measurement method can provide the basis for biomechanical studies investigating the role of tibial torsion with state-of-the-art techniques, such as movement and gait analysis.

## Conclusion

The novel 3D measurement technique shows a high interobserver agreement and offers an applicable opportunity to assess the individual three-dimensional anatomy for assessing tibial torsion. As three-dimensional planning becomes successively more important for many surgical procedures, our measurement technique can provide the basis for the planning workflow in 3D as well as for future studies aiming for improved understanding of the tibial torsion and its role in healthy and pathological biomechanics.

## Data Availability

The datasets used and/or analyzed during the current study are available from the corresponding author on reasonable request.

## Abbreviations

2D: two-dimensional
3D: three-dimensional
CT: computed tomography
CTC: condylar tip clusters
DDH: developmental dysplasia of the hip
DTA: distal tibial axis
DTJP: distal tibial joint plane
ICC: intraclass correlation coefficient
MAD: mean absolute differences
mm: millimeters
MR: magnetic resonance
PCA: principal component analysis
PTA: proximal tibial axis
T: tibia
TF: tibia and fibula
TFCA: tibiofibular contact area
TPP: tibial plateau plane
